# Spontaneous Genomic Alterations in a Chimeric Model of Colorectal Cancer Enable Metastasis and Guide Effective Combinatorial Therapy

**DOI:** 10.1371/journal.pone.0105886

**Published:** 2014-08-27

**Authors:** Yinghui Zhou, William M. Rideout, Angela Bressel, Sireesha Yalavarthi, Tong Zi, Darren Potz, Samuel Farlow, Joelle Brodeur, Anthony Monti, Shailaja Reddipalli, Qiurong Xiao, Steve Bottega, Bin Feng, M. Isabel Chiu, Marcus Bosenberg, Joerg Heyer

**Affiliations:** 1 Department of Research, AVEO Pharmaceuticals, Inc., Cambridge, Massachusetts, United States of America; 2 Department of Dermatology, Yale University School of Medicine, New Haven, Connecticut, United States of America; Cedars-Sinai Medical Center, United States of America

## Abstract

Colon cancer is the second most common cause of cancer mortality in the Western world with metastasis commonly present at the time of diagnosis. Screening for propagation and metastatic behavior in a novel chimeric-mouse colon cancer model, driven by mutant p53 and β-Catenin, led to the identification of a unique, invasive adenocarcinoma. Comparison of the genome of this tumor, CB42, with genomes from non-propagating tumors by array CGH and sequencing revealed an amplicon on chromosome five containing CDK6 and CDK14, and a KRAS mutation, respectively. Single agent small molecule inhibition of either CDK6 or MEK, a kinase downstream of KRAS, led to tumor growth inhibition in vivo whereas combination therapy not only led to regression of the subcutaneous tumors, but also near complete inhibition of lung metastasis; thus, genomic analysis of this tumor led to effective, individualized treatment.

## Introduction

Colorectal cancer (CRC) is one of the four most common and lethal tumor types causing approximately 53,000 deaths annually in the US alone. Fortunately, progress is being made against CRC as both the incidence and mortality from this tumor type have been declining over the past decade at approximately 2–3% per year. Despite this general progress, the average five year survival rate for stage 4 metastatic colorectal cancers is still a grim 12.5% [1]. Many genetic alterations that contribute to CRC have been identified including mutations which inactivate tumor suppressor genes (e.g. p53 and APC mutated in 52% and 76% of tumors, respectively) or activate oncogenes (e.g. KRAS and BRAF mutated in 42% and 10% of tumors, respectively) [2], [3]. Patients with stage 3 or 4 CRC need more effective and less toxic treatments to improve their survival and quality of life. As more targeted therapeutics become available, clinical trials have revealed that many of them are only modestly effective when used as a single agent. In contrast, some show significantly better outcomes when combined with existing chemotherapeutics or other targeted drugs [4], [5]. In order to more accurately screen drug combinations for those that will provide the greatest benefit, accurate and predictive models are needed. Genetically engineered mouse models of CRC have been developed over the past two decades principally by the inactivation of tumor suppressor genes (e.g. APC1638N, [6] for review see [7]). Existing preclinical models of CRC fall primarily into four categories: established cell line xenografts, patient derived xenografts (PDX), germline genetically engineered mouse models (GEMMs) and Cre-Lox conditional GEMMs. Each of the model systems has its own set of advantages and disadvantages with cell line xenografts being the easiest to perform but most distantly related to the primary tumor in the host, while at the other end of the spectrum Cre-Lox activated GEMMs arise in the desired tissue in an immunocompetent host.

The recent collection and propagation of tumors directly from patients has yielded new PDX resources which maintain greater tumor heterogeneity, both histologically and genetically [8]. However, establishment of human tumors in immunocompromised mice exerts selective pressure both in the adaptation to a mouse host and usually to an ectopic site (i.e. subcutaneous). This inherently constrains the array of interactions that normally occur between the tumor, the stroma and the hematopoietic system. In several studies the propagation efficiency of patient-derived material in immunocompromised mice has been mixed, with reports of up to 77% of very advanced human colon tumors propagating in nude mice [9].

GEMMS overcome many of the limitations of xenografts. Current GEMMs of colon cancer develop tumors in a short time frame driven by characteristic mutations in crucial oncogenes and tumors suppressor genes (for review see [7]). Some existing mouse models of colon cancer, which employ relevant RAS mutations, have provided insight on potential drug responses [10]. Our approach to creating GEMMs involves a non-germline, mouse chimera based system with which we have generated complex models of cancer, including breast and lung models [11], [12]. Our embryonic stem cell approach allows the rapid generation of new doxycycline inducible models with multiple genetic modifications without having to cross breed the modified alleles together. Tumors from these model systems have been propagated as allografts and used to generate archives of tumor material thus permitting the prospective examination of drug response and the correlation of that response to tumor characteristics [12], [13]. Molecular characterization by microarray and array comparative genomic hybridization (aCGH) analyses of such propagated libraries of tumors have demonstrated that additional tumor promoting mutations had occurred in the tumor lines affecting their growth rates, metastasis, and response to therapeutics [12], [13], [14]. While a range of biomarker gene expression screens are becoming increasingly useful in directing therapy in ongoing clinical trials (e.g. 21, 50, 70 candidates respectively for Oncotype Dx, PAM50, and Mammaprint [15]) and next generation sequencing of known cancer genes (n = 236) is starting to direct beneficial selection of therapy for a broad array of tumor types [16], the increasing affordability of genome wide screening of expression, copy number, and sequence may provide an unbiased approach to determining effective cancer therapy.

In this paper we describe the process of making an ES cell based model of colon cancer driven by p53 mutation, and inducible activated β-Catenin (*CTNNB1*). *CTNNB1* mutations are found in 5% of human CRC with and without corresponding mutations in *APC* [2] especially in hereditary nonpolyposis colorectal cancer (HNPCC) patients where the frequency of *CTNNB1* mutation rises [17]. The tumors that arise from these ES cell based models contain identical genetic background and initial driver mutations; however, they tend to acquire de novo mutations and copy number variations during tumorigenesis that create limited diversity which can influence response to treatment. The β-Catenin (ΔN131) -driven model we present here generated novel insights into genetic alterations necessary for mouse tumor propagation, metastasis, and drug response.

## Results

### ES cell based chimera colon model

**Figure 1:**
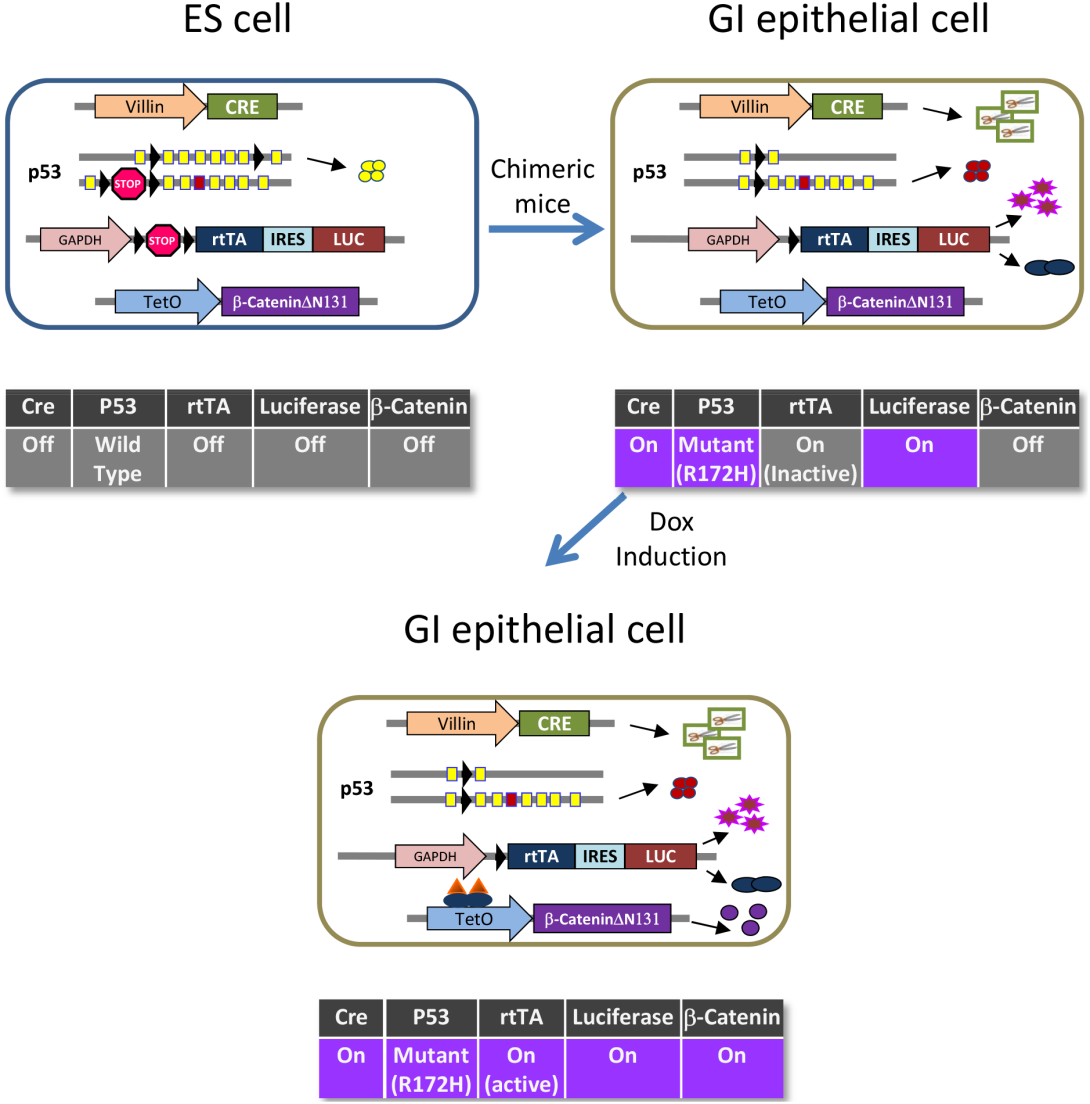
Schematic representation of genetic engineering of the β-Catenin-driven chimeric colon model. A). In ES cells: 3 transgenic cassettes were inserted: Villin-Cre, GAPDH-lox-stop-lox-rtTA-IRES-Luciferase, and TetO- β-CateninΔN131; all three remain silent in ES cells. In addition, one p53 allele was floxed and expresses wild type p53 in ES cells and the other allele was targeted with lox-stop-lox and R172H point mutation, which is functionally null in ES cells. B). In the gastro-intestinal epithelium, expression of Cre leads to deletion of the two lox-stop-lox cassettes as well as the floxed p53 allele. p53 expression will switch from wild type to the mutant form, and expression of rtTA and Luciferase will switch on. C). in the presence of doxycycline, rtTA will bind to the TetO promoter and activate transcription of β-CateninΔN131.

We previously reported a novel approach for modeling cancer development in mice using ES cell chimeras. Compared with the conventional germline transgenic models, one of the key advantages of the chimeric approach is that it allows cells harboring oncogenic mutations to coexist with neighboring cells that are genetically wild type, better mimicking the oncogenic process in human cancers. Deletion of APC, a known tumor suppressor, is a key event in colon cancer progression. Inactivation of APC results in elevated nuclear β-Catenin levels, which prevents terminal differentiation and promotes proliferation. To generate colon model ES cell lines, we started with an Ink4a +/− line (H12C23) and sequentially modified the two p53 alleles: the first p53 allele was made conditional by inserting loxP sites flanking exons 2 to 7 which changes from a functional allele to a null allele upon Cre mediated recombination; the second p53 allele has a lox-stop-lox cassette inserted into intron 1 combined with the R172H mutation in exon 5 which results in expression of an oncogenic version of p53 after Cre-mediated removal of the stop cassette. Next, a GAPDH-lox-stop-lox-rtTA-IRES-luciferase cassette (the activator/marker cassette) was constructed, which, when activated by Cre, expresses rtTA (reverse tetracycline trans-activator) and Luciferase simultaneously. Finally, the ES cells were co-transfected with constructs for GAPDH-lox-stop-lox-rtTA-IRES-luciferase, Villin-Cre and TetO-β-Catenin (ΔN131), a truncated version of β-Catenin that lacks the region that binds the APC/AXIN complex and constitutively accumulates in the nucleus. In non gastrointestinal epithelial cells of resulting chimeras, the Villin promoter will be silent and thus Cre will not be expressed. In this case, one p53 allele remains wild type and the rtTA activator cassette is inactive (Fig. 1 ES cell). In target cells in the intestinal epithelium where Villin promoter drives Cre expression, only mutant p53 will be expressed and the activator cassette will express rtTA and Luciferase and therefore, β-Catenin(ΔN131) expression can be specifically induced in these cells by doxycycline (Fig. 1 GI cell and GI cell + dox).

Twenty ES clones positive for all five genetic elements were injected into blastocysts to generate chimeras, which were given 2500 ppm Doxycycline food after weaning. After induction for 2–4 weeks, for each ES line, 2–3 chimeras with >80% chimerism by coat color were sacrificed and tissues were collected from 3 different regions of the intestinal tract. We analyzed expression of Cre, rtTA, and β-Catenin by qRT-PCR and recombination of the p53 alleles and the activator cassette by PCR. These analyses identified 5 genetically and functionally validated ES lines (91A2, 91C5, 91D3, 91F5, and 91F7) for further study (Data not shown).

### β-Catenin induction in the colonic epithelium leads to invasive adenocarcinomas

One hundred and twenty-nine chimeric mice were generated using the 5 validated colon model ES cell lines and fed with doxycycline food after weaning. After 3 months of induction, we screened for occult blood on a monthly basis and mice positive for 2 consecutive months were monitored daily and sacrificed when weight loss was observed. The intestinal tract was dissected longitudinally, rinsed in PBS to remove feces, then flattened on a paper towel and scanned under a stereoscope for polyps and tumors.

**Figure 2:**
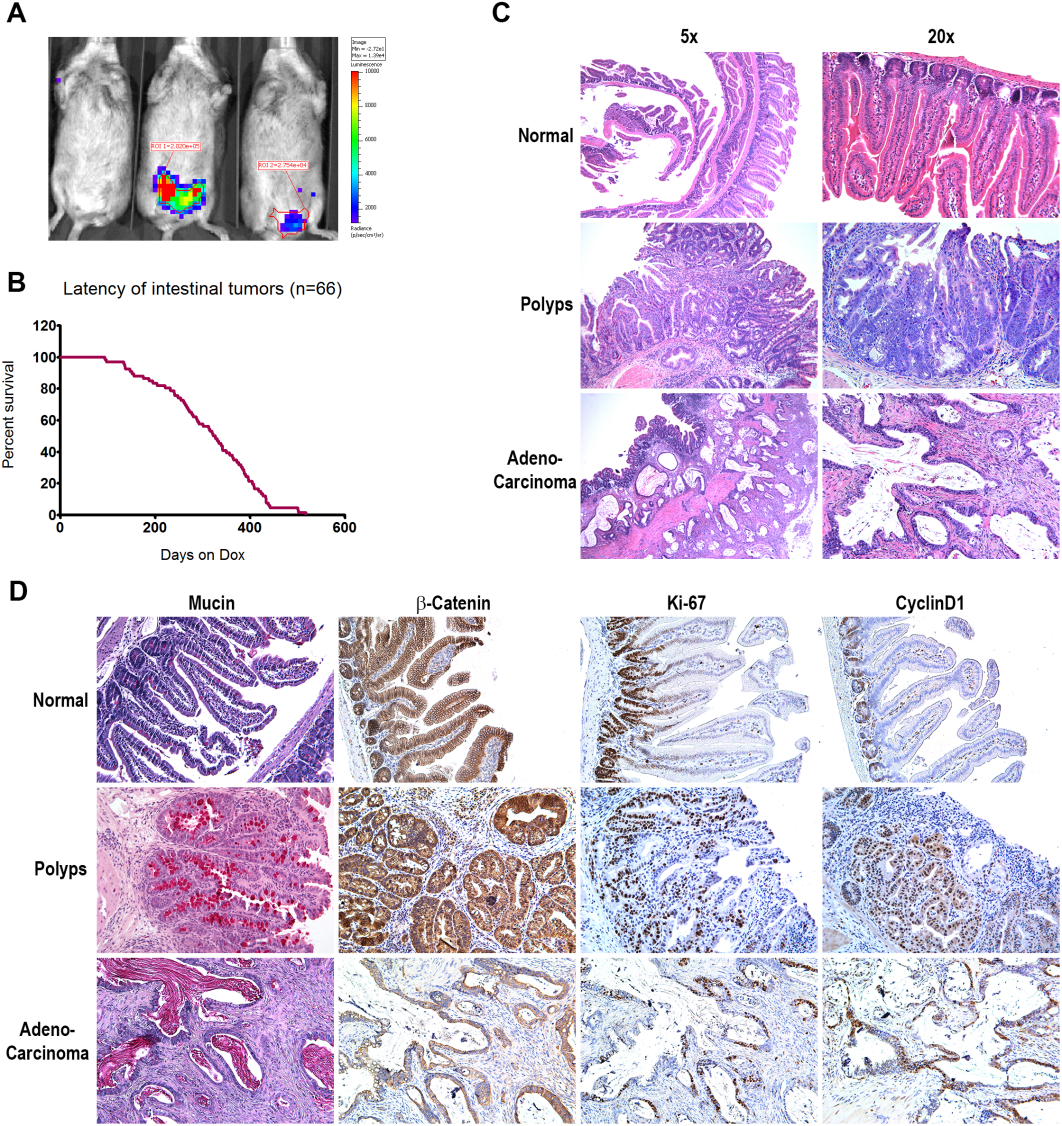
Characterization of the β-Catenin-driven chimeric colon model. A). Bioluminescent imaging of three chimeric mice after three months of doxycycline induction. Luminescent signals were detected in the lower abdominal region in two mice. B). Kaplan-Meyer curve showing the latency of colon tumor development in chimeric mice. C). Histological comparison of normal gastrointestinal tract, benign polyps, and adenocarcinoma from chimeric mice. D). Chemical colorimetric staining for Mucin and immunohistochemical staining for β-Catenin, Ki-67, and Cyclin D1 in normal GI epithelium, polyps and adenocarcinoma.

Fourty-four percent of chimeric mice induced with doxycycline developed intestinal neoplastic lesions within 3–17 months with a median tumor latency of 11 months (Fig. 2B, and Table 1). Bioluminescent imaging demonstrated increased Luciferase activity in the lower abdominal region of tumor bearing mice (Fig. 2A). In these mice, we identified on average 2–3 neoplastic lesions per mouse; histological analysis confirmed 60% of the lesions as polyps, 25% as adenomas and 15% as adenocarcinomas (Fig. 2C). Interestingly, the early lesions (polyps and adenomas) were found equally in both the upper and lower intestinal tract whereas the vast majority (85%) of advanced lesions were found in the colon. The adenocarcinomas ranged from 2–8 mm in diameter and resembled human colon tumors histologically. Invasion into the sub-mucosal layer was observed in 70% of the adenocarcinomas (Fig. 2C). These results demonstrated that nuclear β-Catenin, in combination with p53 mutation, was sufficient to drive malignant tumor development in the GI tract.

**Table 1:** Penetrance and latency of 5 colon model ES cell lines.

	Penetrance			Latency (months)		
ES_Line	Scanned	Positive	%	Median	Min	Max
91A2	18	7	39%	12.1	4.6	13.6
91C5	70	37	53%	10.0	3.1	17.3
91D3	15	4	27%	11.0	10.4	16.7
91F5	16	5	31%	12.5	9.2	13.9
91F7	10	4	40%	10.2	3.2	14.2
**All**	**129**	**57**	**44%**	**10.9**	**3.1**	**17.3**

### Molecular characterization of β-Catenin driven colon tumors

Analysis by immunohistochemistry of candidate proteins in the β-Catenin-driven primary tumors indicated the activation of several pathways known to play a role in colon tumorigenesis. The normal colonic epithelium forms well organized crypt-villus units (Fig. 2C), with dividing progenitor cells located in the crypt region and differentiated non-dividing cells in the villus as evidenced by the restriction of Ki-67 and Cyclin D1 positive cells to the crypt. While membranous β-Catenin is detected on all epithelial cells, nuclear β-Catenin is found only in 20–30% of the cells within the crypt (Fig. 2D). In contrast, the normal crypt-villus structure was lost in both polyps and invasive adenocarcinomas. β-Catenin staining was no longer restricted to the membrane; instead 80–90% of the cells exhibited cytoplasmic and nuclear β-Catenin positivity. 60–80% of the tumors cells showed strong Ki-67 staining and Cyclin D1 positivity, demonstrating that the tumor cells were actively proliferating. In addition, although strong positive staining for epithelial Mucin was detected in 20–40% of cells in all benign polyps studied, consistent with their colonic epithelial origin, Mucin was not detected in the adenocarcinomas examined (Fig. 2D). A fraction of the adenocarcinomas also showed over expression of EGFR (Data not shown).

### Serial propagation of colon tumors

The long latency of this model, in conjunction with asynchronous tumor development, presented a hurdle to effectively carry out preclinical therapeutic studies in a statistically meaningful way. To overcome that problem, we explored ways to expand the tumor material collected from this model through serial in vivo propagation. So far, reports of mouse colon tumor propagation have been absent from the literature. We tested in vivo propagation in 3 different strains of immunocompromised mice, (SCID, NOD-SCID and Nude) in 3 different sites (subcutaneous space, kidney capsule, and cecal sub-mucosal space) (see Table S1 for details) of either tumor fragments or single cell suspensions embedded in Matrigel. Propagation of tumor material from 82 tumors was tested in a total of 107 sites. Of the tumors tested, only one successfully established a propagated tumor line, CB42, which arose in the cecum of a chimeric mouse from the ES line 91C5 (Table 2 and Table S1).

**Table 2:** Colon model tumor propagation statistics.

Propagation Method	Primary tumor	Passage 1	Passage 2
SQ Implantation	40	4	0
SQ Injection	12	1	1 (CB42)
Kidney Capsule Implantation	32	3	0
Kidney Capsule Injection	14	1	1 (CB42)
Cecal sub-mucosal Injection	9	1	0

**Figure 3:**
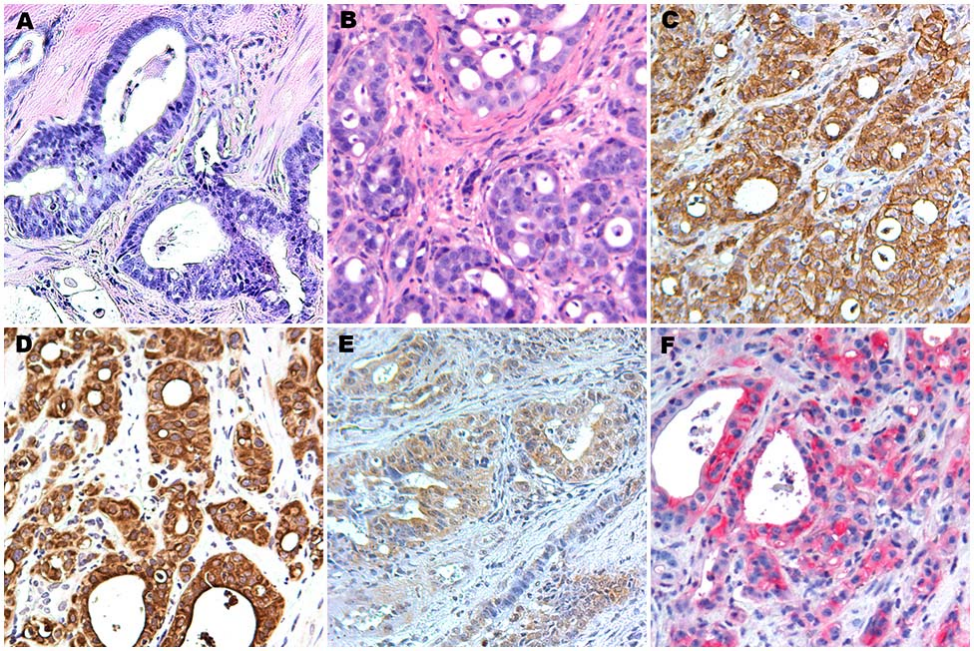
Characterization of CB42. A) Histology (H&E staining) of CB42 primary tumor. B). Histology (H&E) of CB42 after subcutaneous propagation (CB42P1). C). Immunohistochemical staining for β-Catenin. D). Immunohistochemical staining for pan Cytokeratin. E). Immunohistochemical staining for HGF. F). Immunohistochemical staining for phospho-Met.

CB42 grew robustly in both the kidney capsule and the subcutaneous space; for example, when 10^5^ cells were injected per site, tumors became visible in 5–7 days and reached humane limits within another 2 weeks. Histological analysis showed that the architecture of tumors propagated either in kidney capsule or the subcutaneous space resembled that of the primary tumor (Fig. 3A, B and data not shown). The propagated tumors retained greater than 80% of cells exhibiting cytoplasmic and nuclear β-Catenin expression (Fig. 3C) and kept their epithelial characteristics as evidenced by homogeneous IHC staining for pan cytokeratin (Fig. 3D) and E-cadherin (data not shown). Immunohistochemical staining for HGF was positive in 70–80% of these tumor cells, and staining for phospho-MET revealed high MET expression and activation, but curiously no EGFR positivity (Fig. 3C–F and data not shown) as commonly seen in human CRC [18] and other primary tumors of the ES-chimera B-catenin colon model.

### Tumor line CB42 metastasizes to liver and lung

**Figure 4:**
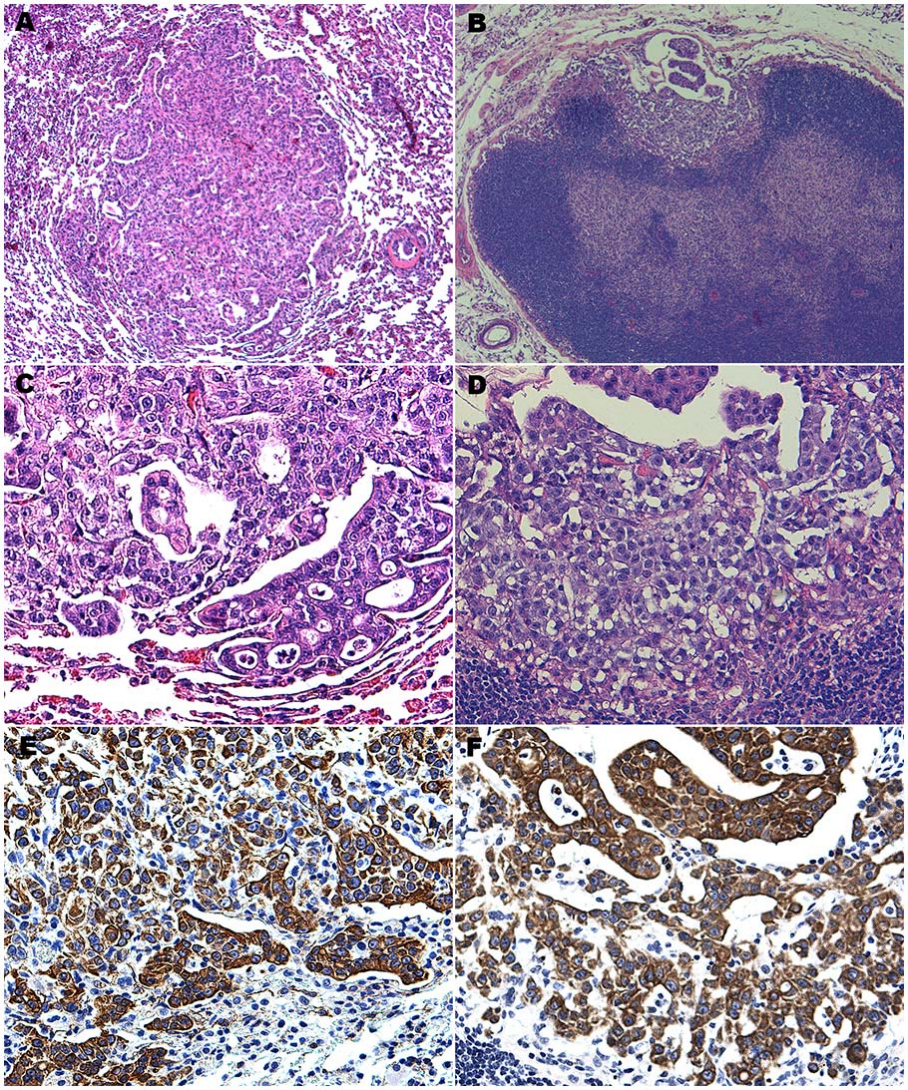
Metastasis of CB42 from subcutaneous space to lung and lymph nodes. Lung metastasis H&E at 5X (A) and 20X (C) objective magnification; lymph node metastasis H&E at 5X (B) and 20X (D) objective magnification; pan-cytokeratin immunohistochemistry of lung (E) and lymph node (F) metastases.

Metastasis presents the real therapeutic challenge in CRC. To validate our CB42 model we studied its metastatic potential. We first carried out 2 seeding assays: seeding of tumor cells in the lung through intravenous injection or in the liver through intrasplenic injection followed by splenectomy. The success rates for both seeding assays were high: in the lung assay, 10 out of 10 mice developed tumors within 4–6 weeks after injection resulting in increased average lung weights for these mice to 1.5 g (compared with 0.2 g for normal lung); in the liver assay, 7 out of 10 mice developed visible tumor nodules in the liver 5–8 weeks after injection. A second series of metastasis experiments more closely recapitulated the process of metastasis in human cancer patients. Tumor cells were first injected into the subcutaneous space. Once the inoculated tumors reached 500–800 mm^3^ in size, we excised them surgically and monitored the mice closely for general healthy appearance and weight loss, an indication of metastasis to internal organs. Eight out of 10 mice showed no sign of regrowth of the subcutaneous tumor and 2 had only a very small nodule (<200 mm^3^) at the surgical site. However, all 10 mice became ill within 3–5 weeks after the surgery. Necropsy revealed metastasis to the lung in all 10 mice and distant lymph node metastasis in 6 of the 10 mice. Careful histological examination of other internal organs including brain, liver, spleen, and kidney did not reveal any tumor nodules (data not shown). The lung and lymph node metastases resembled the primary tumor histologically, and were positive for pan cytokeratin staining (Fig. 4). It was not clear however, based on this experiment, whether the surgery was causally linked to tumor cell dissemination to distant organs, or merely allowed the disseminated tumors more time to grow in the distant organ by prolonging the life of these mice. To distinguish these two possibilities, we repeated the experiment without the surgical removal of the subcutaneous tumor. The mice were sacrificed when the subcutaneous tumors reached humane limits and carefully examined for signs of metastasis. Small whitish nodules were found in the lungs of 5/10 mice, and 2 of these mice had swollen ancillary lymph nodes near the neck. This experiment demonstrated that CB42 was competent to metastasize spontaneously

### Spontaneous gene copy number alterations in CB42

**Figure 5:**
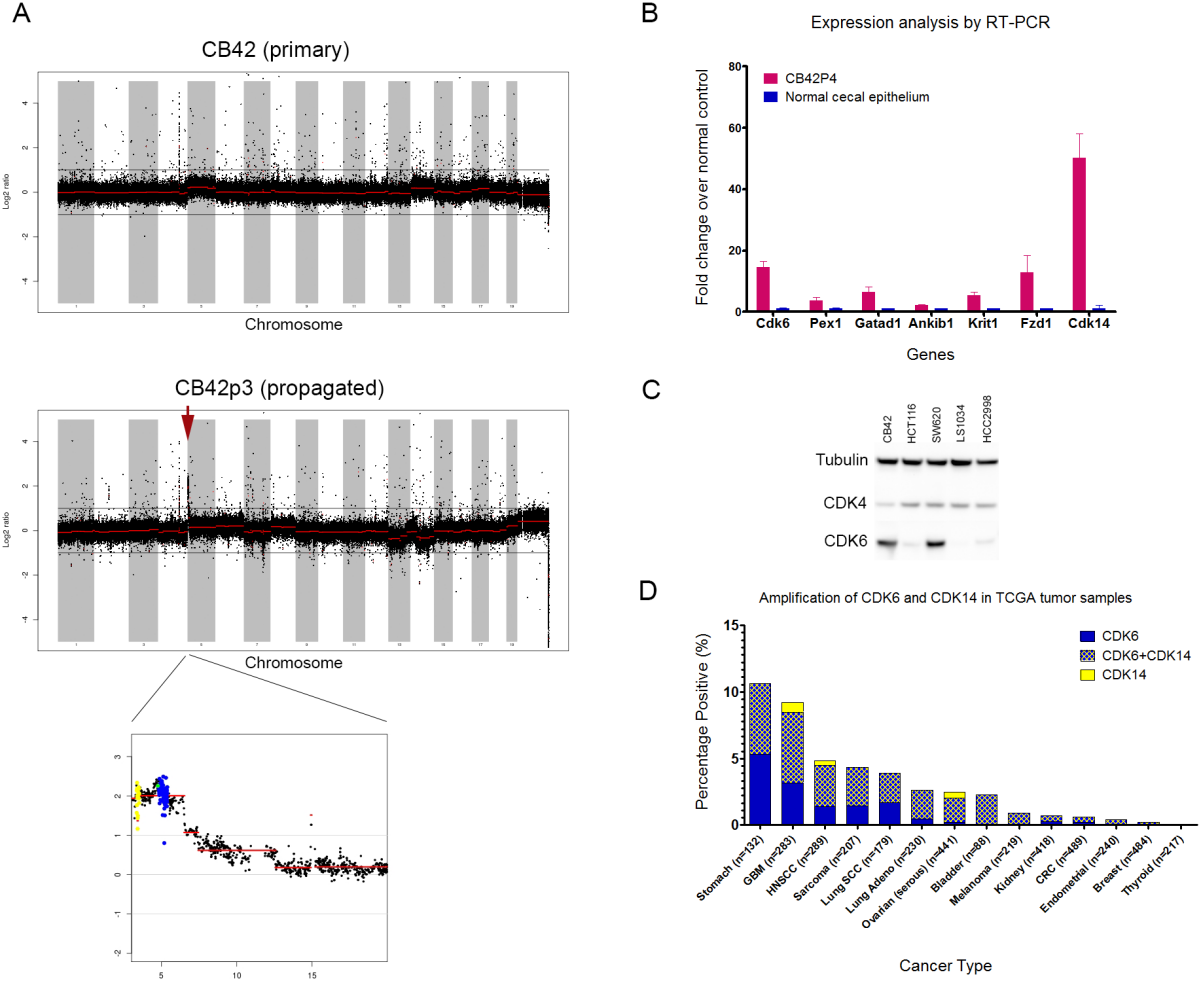
Amplification of Cdk6 and Cdk14 in propagated CB42 tumors. A). aCGH profile of CB42 primary tumor and passage 3 propagated tumor. A 3.5 Mb amplicon on chromosome 5 was detected only in the propagated tumor. Probes for Cdk6 were colored in yellow and probes for Cdk14 were colored in blue. B). RT-PCR analysis for the expression of 7 genes in the chromosome 5 amplicon. The relative expression values of CB42P4 tumors (n = 4) were normalized to those of normal cecal epithelium (n = 4). C). Western Blot analysis for levels of Cdk4 and Cdk6 in CB42 and 4 human colon cancer cell lines. D). Frequency of CDK6 and CDK14 amplification in human cancer samples from 13 tissue types. Data were retrieved from CBio website.

Since CB42 was unique among our β-Catenin driven colon tumors in its ability to propagate and metastasize, we performed a series of unbiased genomic assays including arrayed comparative genomic hybridization (aCGH) and sequencing to identify spontaneous genomic alterations that correlated with propagation and metastasis. Genomic DNA isolated from CB42 primary and subcutaneous, serially-propagated (passage 3, labeled as CB42P3) tumor samples along with DNA from 7 other primary tumors that did not propagate were chosen for comparison by aCGH. Analysis of the aCGH data revealed multiple amplicons that were present in only the subcutaneously propagated CB42 tumors (CB42P3), but not the primary CB42 tumor, nor any of the other primary colon tumors (Fig. 5A).

The most prominent amplicon was a 3.5 Mbp region on Chromosome 5 between Cdk6 and Steap1 containing more than 25 known genes. We examined by qRT-PCR the expression of 7 candidate genes within the amplicon and discovered that 3 of the 7 genes were significantly overexpressed in comparison with normal GI tract epithelium: Cdk6, Cdk14 (Pftk1) and Fzd1 (Fig. 5B). Western blot analysis of CB42 and several human colon tumor xenograft lines confirmed high expression of CDK6 in CB42 and SW620 and detectable CDK6 in HCT116 and HCC2998 indicating that activation of CDK6 may be advantageous for human tumor xenografts (Fig. 5C). Analysis of the Cancer Genome Atlas (TCGA) database found evidence for amplification of the syntenic region in the human genome and revealed that while amplification of the region was uncommon in human CRC, it ranged up to 11% of gastric tumors (Fig. 5D).

### Mutations identified by genome sequencing

In addition to copy number changes, we examined DNA from propagated CB42P3 tumors for mutational changes by whole genome, exome, and RNA sequencing which consistently identified 9 mutations in exon regions (Table 3). One of the mutations caused a change of Glycine to Cysteine at the codon 12 position of the KRAS gene. Subsequent pyro-sequencing of DNA from both primary and propagated CB42 as well as 10 other primary colon tumors confirmed the KRAS mutation only in propagated CB42, but found no KRAS mutations in any of the other samples, thus suggesting that activation of KRAS was not required for primary colon tumor initiation in the CRC model, but was required for malignant growth of the tumor during propagation and metastasis.

**Table 3:** List of mutations identified in CB42P3 by next gen sequencing.

Chr:Position	Transcript ID	Gene Name	NucleotideChange	Amino AcidChange	MutationFrequency	COSMICcancer genecensus
11∶75506133	NM_133656	Crk	c.676G>A	p.Val226Ile	39.3%	
11∶86898884	NM_024262	1200011M11Rik	c.1372G>A	p.Val458Met	61.5%	
16∶28828891	NM_177718	1600021P15Rik	c.416G>A	p.Arg139His	46.2%	
18∶6208228	NM_008448	Kif5b	c.2867G>C	p.Arg956Pro	37.9%	Yes
6∶145195292	NM_021284	Kras	c.34G>T	p.Gly12Cys	59.6%	Yes
6∶52687105	NM_025816	Tax1bp1	c.791A>G	p.His264Arg	31.0%	
7∶134924067	NM_178029	Setd1a	c.415C>T	p.Arg139Cys	33.3%	SETD2
7∶148462370	NM_001114322	Cdhr5	c.170T>A	p.Val57Asp	50.9%	
7∶29620934	NM_010706	Lgals4	c.223G>A	p.Val75Ile	73.3%	

### Growth and Metastasis of CB42 is MET/HGF pathway independent

**Figure 6:**
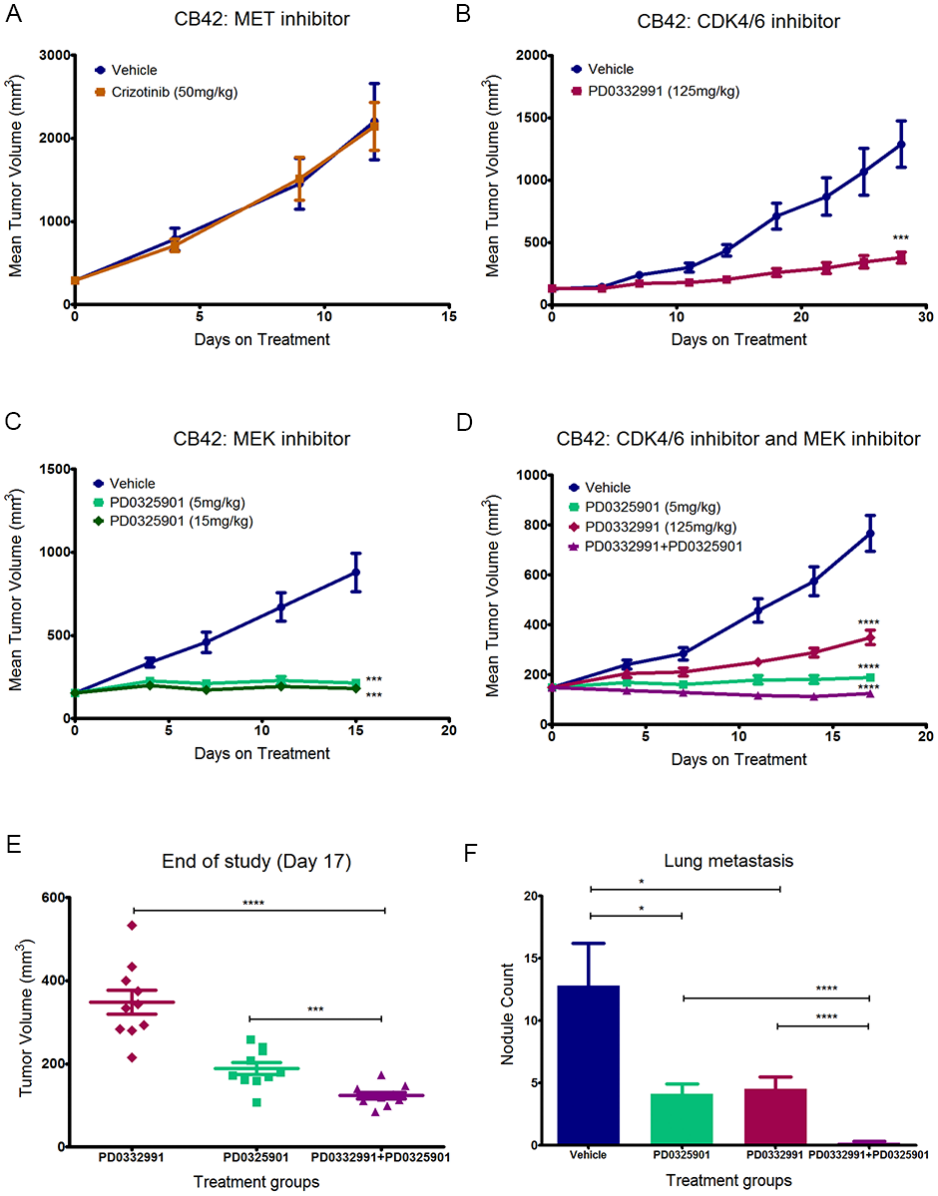
Treatment of CB42 subcutaneous allografts (n = 10 per treatment arm) and subsequent metastasis to the lung in nude mice with targeted therapy. A). MET inhibitor crizotinib did not inhibit CB42 tumor growth. B). CDK4/6 inhibitor PD0332991 significantly inhibited CB42 tumor growth (***P<0.001). C). MEK1/2 inhibitor PD0325901 completely blocked CB42 tumor growth (***P<0.001). D). Combination of CDK4/6 and MEK1/2 inhibitor induced CB 42 tumor regression (****P<0.0001). E). Subcutaneous tumor volumes of the three treatment arms at the end of study. The combination is significantly more potent than CDK4/6 inhibitor alone (****P<0.0001) or the MEK inhibitor alone (***P<0.001). F). Gross examination of lung lobes for metastatic nodules revealed that both CDK4/6 inhibitor and MEK inhibitor significantly inhibited CB42 lung metastasis (*P<0.05), but the combination was significantly more effective than either agent alone (****P<0.0001) and almost completely eliminated lung metastasis.

Since MET expression and activation were elevated in propagated CB42 (Fig. 3F) and the role of MET in invasion and metastasis is well supported in the literature [19], [20], [21], we tested whether the MET pathway might play an important role in CB42 proliferation and metastasis. To test this hypothesis, we carried out a series of 3 experiments. In the first experiment, we treated mice implanted with subcutaneous CB42 tumors with crizotinib (50 mg/kg po qd), a small molecule inhibitor of MET and ALK. Surprisingly, despite the high level of MET expression and activation, treatment of CB42 with crizotinib had no effect on tumor growth (TGI = 0%, Fig. 6A). In the second experiment, we studied whether crizotinib could inhibit CB42 metastasis. CB42 tumor cells were injected subcutaneously and the primary tumors were surgically removed after 14 days, when the average tumor size reached 500 mm^3^. These mice were cohorted into one vehicle group (n = 10) and one treatment group (n = 10), and treatment started the second day after surgery. Mice in both groups started to become ill 2–3 weeks after surgery. When they were sacrificed, lung metastases were found in 9/9 mice in the vehicle group and 10/10 mice in the crizotinib treated group, while lymph node metastasis was observed in 2 mice from each group. No significant difference in numbers or size of metastases was observed between treatment groups, suggesting that inhibition of MET did not influence metastatic growth after establishment of distant metastatic tumors.

In the third experiment, we tested whether MET inhibition could prevent tumor cell dissemination out of small primary tumor sites. In this experiment, dosing with vehicle or crizotinib started two days after subcutaneous injection of CB42 cells into recipient mice. The subcutaneous tumors were surgically removed two weeks later and the dosing continued until the mice became sick. Lung metastases were observed in all the mice, regardless of whether they received crizotinib or vehicle. Based on these experiments, we concluded that MET was not required for CB42 tumor growth and metastasis.

### Significant inhibition of subcutaneous and metastatic tumor growth by CDK4/6 or MEK inhibition

Since propagated CB42 had a genomic amplification of CDK6 and strong cellular expression (see above), we tested a compound, PD0332991, which inhibits both CDK4 and CDK6 [22]. Despite the confirmation by Western blot that showed strong CDK6 and weaker CDK4 expression in CB42 (Fig. 5C), a 3 day in vitro proliferation assay showed that PD0332991 did not inhibit proliferation of a CB42-derived cell line even at the highest dose (10 µM) (data not shown). In contrast, treating mice implanted with subcutaneous CB42 tumors with PD0332991 (125 mg/kg, po qd) strongly inhibited subcutaneous tumor growth (TGI-75%, p<0.00005, Fig. 6B) and reduced lung metastasis. At the end of the experiment, only five out of 15 mice treated with PD0332991 had visible nodules in the lung whereas all 15 mice in the vehicle groups had visible lung metastases. There were on average 5 lung nodules per mouse in the PD0332991 group vs. 18.3 in the vehicle group (p = 0.00002). Overall these results suggested that CDK6 overexpression allows mouse GI tumors to grow outside the colonic sub-mucosal environment and that activation of CDK6 might contribute to GI tumors surviving and thriving in a metastatic setting.

Since CB42 had also acquired an activating Kras mutation and literature suggested that KRAS mutant tumors were sensitive to MEK inhibitors [23], the MEK inhibitor PD0325901 was tested for its ability to inhibit the growth of CB42 in vitro and in vivo. MEK inhibition blocks downstream signaling of KRAS. In vitro, PD0325901 potently blocked Erk1/2 signaling in CB42 cells (data not shown). In vivo, a single dose of PD0325901, either at 5 mg/kg, or 15 mg/kg, resulted in complete inhibition of Erk1/2 phosphorylation 4 hours after dosing (data not shown). Treatment with PD0325901 of subcutaneous CB42 tumors in mice, at either 5 mg/kg or 15 mg/kg, lead to near complete tumor growth inhibition (93% and 96% deltaTGI, respectively, Fig. 6C). Scanning of the lungs at the end of study also demonstrated a significant reduction of both number and size of metastatic nodules in the treated mice vs. vehicle group. These data demonstrated that the growth and metastasis of CB42 as a subcutaneous allograft was also strongly dependent on the KRAS mutation.

### Regression of CB42 by combined inhibition of KRAS/MEK signaling and CDK4/6

The multiple genetic lesions identified in CB42 suggested that maximum therapeutic effect might be achieved with a combination of targeted agents that inhibit several of the dysregulated pathways. To test this hypothesis, we treated CB42 with the MEK inhibitor PD0325901 (5 mg/kg) in combination with the CDK4/6 inhibitor PD0332991 (125 mg/kg). The combination of these two drugs was well tolerated and did not result in significant weight loss (<10%) (Data not shown). Combined treatment with PD0332991 and PD325901 did dramatically regress tumors by an average of 35% (Fig. 6D), while the single agent treatment arms again showed significant tumor growth inhibition compared to controls. Comparison of the individually plotted tumor sizes between the three treatment arms at the end of study illustrated how significant and uniform tumor regression was in the combination arm (Fig. 6E). Also at the end of study, the lungs were dissected and examined for observable metastatic nodules. Fewer metastases were observed in both single agent treatment arms (down to 5 nodules/mouse compared to 15 nodules/mouse in the vehicle control arm), while notably, the combination arm averaged less than one nodule per mouse (Fig. 6F). Thus, for CB42, both acquired genetic lesions contributed to subcutaneous tumor growth as well as metastatic potential and/or metastasized tumor growth.

### Enforced expression of CDK6 and CDK14 resulted in propagatable colon tumors

Although there is some literature evidence that both CDK6 [24], [25] and CDK14 [26],[27] are involved in various aspects of tumorigenesis, their potential roles in colon tumorigenesis are currently not well understood. We took advantage of the flexibility of the ES chimera model approach and added doxycycline-inducible expression cassettes of CDK14 alone or in combination with CDK6 and Fzd1 to the original colon model ES line 91C5 to establish new model lines. Chimeric mice generated from these lines were induced with doxycycline food and observed for tumorigenesis.

The addition of the new transgenes did not significantly affect tumor latency (8–12 months). Interestingly, the majority of the tumors (3 out of 4 tested) expressing either CDK14 alone or all three transgenes could be propagated subcutaneously; hinting that CDK14 alone could be sufficient to permit propagation.

**Figure 7:**
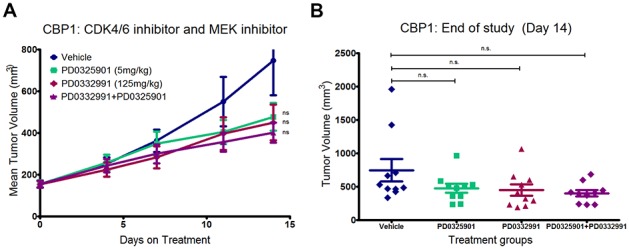
CBP1, which lack Cdk6 amplification and KRAS mutation, did not respond to either the CDK4/6 inhibitor or the MEK inhibitor. The mild tumor growth inhibition is not statistically significant from the vehicle for all three treatment arms (P>0.05).

One propagated tumor line, CBP1, from a model ES cell line overexpressing CDK14 in addition to β-Catenin was tested to assess whether a propagatable colon tumor lacking KRAS activation and CDK6 amplification would respond to either of the targeted agents or their combination. In contrast to CB42, CBP1 showed a non-significant trend toward mild tumor growth inhibition in response to the MEK-inhibitor, the CDK6-inhibitor, or the combination, reaching at most 45% TGI in the combination arm (Fig. 7A). However, end of study tumor volumes further reinforced the assessment that CBP1 had no significant response to the MEK and CDK6 inhibiting drugs (Fig. 7B). Taken together, the data from the drug treatments of CB42 and CBP1 strongly demonstrated that the mutation and amplification events in CB42 were directly responsible for the exquisite sensitivity to each of the drugs and more dramatically, to their combination. These results suggest that CDK6 and CDK14 play a crucial role in generating mouse colon model tumors which are able to grow and metastasize away from their site of initiation.

## Discussion

We have recently introduced a novel approach to modeling cancer in mice utilizing chimeric mice [11]. Here we report a complex mouse model of colon cancer which has been established utilizing this chimera approach, in which genetically modified ES-cells (p53-flox, p53-loxSTOPlox-R172H, GAPDH-loxSTOPlox-rtTA, and Villin-Cre) were engineered to express activated inducible β -Catenin (ΔN131) and mutated endogenous p53 (R172H) in the mouse gastrointestinal epithelium. The tissue-specific, inducible nature (tet-on) of the β-Catenin oncogene combined with Cre-Lox alteration of the p53 tumor suppressor gene led to the development of invasive gastrointestinal adenocarcinomas; though some chimeras developed lymphomas and sarcomas due to the heterozygous mutations of p53 and Ink4a present in the ES cells. This mouse tumor model resembles human colorectal tumors which commonly harbor mutations that activate Wnt signaling, generally through common mutations in APC and occasionally β-Catenin [3], [28]. These mutations allow β-Catenin protein to escape cytoplasmic retention by the APC complex [29]. The function of the β-Catenin mutations has not yet been elucidated when compared to the well-characterized APC deletions and mutation in human and mouse. Here we demonstrated that β-Catenin activation in the mouse GI tract can lead to tumor formation. Our observations hint that this could be due to an increased proliferation readiness in the stem cell compartments rather than overall proliferation of the whole epithelium since no aberrant epithelial structures were detectable upon β-Catenin induction.

As observed in other mouse models of gastrointestinal cancer, the CB mouse model develops invasive adenocarcinomas [10], [29], [30], [31]. Again as with the other models, we do not observe any metastasis in primary tumor bearing animals, an observation that distinguishes mouse models from human colon tumors that readily metastasize to the liver and lung (the incidence of distant metastasis at time of diagnosis is 32.5% in US men [32]).

Human colorectal tumors are frequently capable of propagating in mice [33] something not yet observed with mouse gastrointestinal tumors. From the β-Catenin-driven model, we identified one mouse tumor that could propagate in immunocompromised mice. Interestingly, the ability to propagate in an ectopic site (e.g. kidney capsule and subcutaneous) coincided with the ability of CB42 to metastasize to lymph nodes and lung as well as produce metastatic nodules in the liver after injection into the spleen; confirming the capability of CB42 to metastasize to the primary organ involved in CRC metastasis in patients. The observation that CB42 can grow at any site of inoculation suggested that its metastatic behavior is driven by acquired genetic alterations within the tumor cells. The fortuitous propagation/metastatic phenotype of CB42 allowed us to test the validity of a personalized medicine approach [34] to characterization and treatment of the genetic alterations that might promote metastasis and propagation of colorectal cancer. We applied a limited candidate gene approach (EGFR, HGF ad MET by immunohistochemistry), array CGH, and genomic sequencing to compare CB42 to other propagation-incapable, β-Catenin-driven primary colon tumors to identify a series of candidate genes that correlated with propagation/metastasis.

Even though IHC found strong activation of the MET receptor in CB42, treatment of tumors by crizotinib, a MET inhibitor, did not inhibit tumor growth. In contrast, the comparative genomic analyses of CB42 successfully identified propagation-specific mutations that had been acquired spontaneously and were known to play a role in human tumorigenesis (e.g. KRAS-G12C). KRAS mutations are frequently observed in human CRC (42% incidence [2], [28]) and validate our CB42 tumor model as reflective of human disease. Interestingly, the KRAS mutation could not be detected in the primary CB42 tumor suggesting tumor heterogeneity and clonal selection during propagation. Since the primary tumor of CB42 propagated in both kidney capsule and subcutaneous injections, it suggests that tumor cells containing the KRAS mutation were already present in the primary tumor. The dependence of CB42 on mutated KRAS was illustrated by the observed potent and significant tumor growth inhibition when treated with a MEK inhibitor, PD0325901, which has been shown to strongly inhibit KRAS driven cancers [23], [35].

Additional genetic alterations were found by array CGH analysis of CB42 tumor DNA led to the discovery of an amplicon on mouse chromosome 5qA1 specific to the propagation of CB42. This amplicon harbors some 25 genes in a span of 3.5 Mbp and contains several candidate genes relevant to CRC (e.g. CDK6, CDK14 and Fzd1). Intriguingly, while this amplicon occurs infrequently in human colon cancer (1–2%), it is found in many other tumor types at low frequencies rising to a high near 10% in gastric cancer [3]. To test the importance for tumor growth and metastasis of these genes we introduced inducible cDNAs of CDK6, CDK14 and Fzd6 alone or in combination into the parental ES cell line of the β-Catenin driven model, 91C5, and generated chimeric mice. None of the new chimeric mice showed accelerated tumorigenesis or higher incidence of tumors, yet 3 of 4 tumors harboring CDK14 or CDK14/CDK6/FZD1 were easily propagated, strongly suggesting that overexpression of CDK6 and CDK14 are important for ectopic tumor growth and metastasis. Indeed, expression data from paired adjacent normal colonic epithelium and primary tumors from human patients showed a dramatic increase in CDK6 expression, followed by a further increase in metastatic lesions [36]. Intriguingly, since CBP1 could propagate without overexpression of CDK6 and activated KRAS, it suggests that CDK14 (PFTK1) could play an important, independent role in the growth of CRC tumors outside the GI tract and metastasis. Comparative data about the role of CDK14 in cancer is sparse, but authors relate that the over-expression of CDK14 promotes Wnt signaling [37], predicts chemotherapeutic resistance [26], and increases hepatocellular carcinoma motility [27].

Further proof that the 5qA1 amplicon was crucial for the phenotype of CB42 was illustrated by treating propagated tumors with a CDK6 inhibitor (PD0332991), which inhibited tumor growth by 75%. Similarly, CDK4/6 inhibition has been shown to inhibit the proliferation of several colon cancer cell lines in vitro [38], induce regression of a human colon cancer xenograft (Colo-205) [22], and induce regression of multiple myeloma xenografts which specifically overexpress CDK6 [39]. Thus, data from human colon tumors and cell lines indicate that the CDK6 pathway is activated and/or overexpressed without direct amplification of the gene.

Armed with the genetic evidence of mutations involving KRAS and CDK6/CDK14 as well as the response to single agent therapies, we reasoned that treatment with a combination of drugs addressing the key mutations associated with propagation and metastasis in the CB42 should result in tumor regression. Essentially we performed a trial of personalized medicine on a mouse model in which the goal was to identify key mutations and exploit them with targeted, rational drug combinations. The test of this paradigm by combination of the MEK and CDK6 inhibitors led to regression and to nearly a complete blockade of metastasis of CB42; proving that personalized medicine is feasible with current technologies. Interestingly, the combination of inhibitors against the MEK1 and CDK4/6 pathways was synergistic in an inducible NRAS^Q61K^-driven mouse model of melanoma and showed striking in vivo tumor regression [40]. Our data also support the hypothesis that evidence-based analysis of genomic and expression data yield effective insights to select combinations of targeted therapies as opposed to a limited candidate target approach by IHC in which the strong histological staining for MET in CB42 yielded no efficacy in response to a MET inhibitor; though a broader panel of antibodies detecting specific pathway activation might prove equally effective.

Tumors rely on dysregulation of multiple pathways in order to proliferate and survive; an unfortunate, probable consequence of this array of dysregulation is the frequently observed poor clinical response to targeted mono-therapy. Taken together, our data support the notion that some mouse models of human tumors likewise can develop via a multi-step process, with additional mutations selected for at each step, until the tumor reaches full malignancy. Previous GEMM models of colon cancer, although engineered with mutations sufficient to support primary tumorigenesis, have likely not acquired all the mutations necessary for the tumors to reach the advanced metastatic stage seen in many human colon cancers at diagnosis. The propagated CB42 tumor on the other hand, is a rare representation of a murine model for metastatic colon cancer. The ability of CDK6 and CDK14 to foster successful propagation when added to the existing colon cancer model may indicate a lack of Cyclin D [24], [36] activity necessary to grow in an ectopic location; perhaps the microenvironment of the primary tumor provides sufficient stimulation of the pathway to allow proliferation in the orthotopic location. These results coupled with the low level but common amplification of the CDK6/CDK14 region of chromosome 7 observed in several human tumor types and the over-expression of CDK6 in human CRC and CRC cell lines [34] [38] suggests that therapy targeting CDK6/CDK14 activity might be beneficial at suppressing proliferation of primary and metastatic tumors.

## Materials and Methods

### Ethics Statement

All animal work in this study was approved by AVEO’s Institutional Animal Care and Use Committee (IACUC protocol number AP2013-023). Animals were cared for in accordance with AAALAC (Association for Assessment and Accreditation of Laboratory Animal Care) policies and certification. All surgeries were performed under avertin or isoflurane anesthesia, and analgesic (buprenorphine, BID) was administered post surgery for 48 hours to minimize suffering.

### Method of Euthanasia

AVEO IACUC regulations require immediate euthanasia of mice showing one of the three following symptoms: moribund, weight loss of 20% or more, or tumor exceeding 2 cm in the longest dimension. All healthy mice were euthanized at the end of each study. Euthanasia was carried out via CO2 asphyxiation in a plexiglass chamber. The carcasses were disposed of either after rigor set in or after applying cervical dislocation.

### DNA constructs

The p53 conditional targeting vector was constructed by inserting a LoxP-FRT-NeoR-FRT cassette into intron 1 and a single LoxP site into intron 7 of the p53 genomic sequence. The FRT-NeoR-FRT cassette was removed from correctly targeted ES clones using transient expression of FLPe, leaving only the LoxP site in intron 1. The p53 lsl.R172H targeting vector was licensed from MIT [41]. The human β-Catenin(ΔN131) cDNA was generated using high fidelity PCR, cloned into a Gateway compatible TetO expression vector and sequence verified. The Villin-Cre construct was a gift from Dr. Sylvie Robine [42]. Human CDK6, CDK14 and murine Fzd1 cDNAs were purchased from Genecopoeia in ORFExpress vectors. The cDNAs were transferred into pTetO-pA by Gateway LR (Invitrogen) reaction to generate the inducible constructs.

### ES cell manipulation

ES clone H12C23, which was first described in [11], was transfected with the p53 lsl R172H targeting vector. One positive clone with correct targeting of p53 was identified by PCR and verified by Southern blots. This ES line 63A4, which was shown to give rise to robust chimeras, was transfected with the p53 conditional knockout targeting vector. Four clones were identified to have the correct targeting event on the p53 wild type allele. One of the clones, 68H3 was transiently transfected with a circular pCAGGS-FLPe (Gene Bridges) plasmid to remove the selection cassette in the conditional allele. One of the resultant clones, 83F11, was then co-transfected with GAPDH.lsl.rtTA.IRES.Luc, Villin-Cre and TetO-β-Catenin(ΔN131). 42 clones were identified by PCR to be positive for all three constructs, and 20 of them were injected into blastocysts to generate chimeras for further characterization as well as the ability to produce tumors.

### Chimeric mice production

Confluent ES cells cultures were washed twice with PBS and trypsinized. Equal volume of ESCM without LIF was added and cells were triturated several times to make single cell suspension. 5 ml of ESCM-LIF was then added to the cells and the cells were spun down, re-suspended in 4 ml of ESCM-LIF and plated on a 60 mm tissue culture plate and incubated for 30 min to remove feeder cells. Cells in suspension were spun down again and re-suspended in minimal volume for injection. Typically 10–15 ES cells were injected into one C57BL/6 blastocyst (3.5 dpc) and 12–16 blastocysts were transferred into the uterine horns of a pseudo pregnant Swiss Webster female (2.5 dpc). Pups are born 17 days after the transfer and chimeras can be identified by their agouti coat color. Chimeras are housed in a SPF animal facility. Chimeras are weaned 3–4 weeks after birth and ear tagged. Doxycycline was delivered in either water containing 2 mg/mL doxycycline in 10 mg/mL sucrose in dark bottles changed twice weekly or in food pellets containing 2500 ppm of doxycycline.

### Occult blood test

Fresh fecal samples were collected from chimeric mice once every month. Testing for the presence of occult blood was carried out using Hemoccult Brand SENSA Slide Test kit following kit instructions.

### Intestinal scanning

The entire intestinal tract was removed from model chimeric mice and laid on a piece of paper towel. The intestine was cut open longitudinally and rinsed in PBS to remove all fecal contents. The rinsed intestine was then opened flat on a petri dish and scanned under a dissection scope for any protrusions into the lumen. The protrusions identified were dissected out for further analysis or propagation.

### Real time RT-PCR

Real time qRT-PCR was carried out in 384-well format on ABI 7900 HT systems using QuantiTech SYBR green RT-PCR kit (Qiagen). Each sample was analyzed in quadruplicate reactions with 50 ng of RNA used in each 20 µl reaction. *HPRT* was used as loading control. The relative expression of each human oncogene was expressed as fold change over Human Universal Reference RNA (Stratagene), which was calculated using the delta delta Ct method in the SDS 2.1 software.

### Immunohistochemistry

Formalin fixed and paraffin embedded slides were first treated in antigen retrieval buffer at 98C for 15 minutes, and then incubated with primary antibody at 4C overnight or 1 hour at room temperature. The next day, after washing, the samples were incubated with HRP conjugated secondary antibody for 30 minutes. They were then washed and incubated with DAB under close monitoring for color development. The β-Catenin antibody was purchased from BD Transduction Laboratories (Cat# 610153), the Ki-67 antibody was purchased from Biocare Medical (Cat# CRM325), the Cyclin D1 antibody was purchased from Millipore (Cat# 04-1151), the Cyto-keratin antibody was purchased from Dako Cytomation (Cat# Z0622), the HGF antibody was purchased from R&D Systems (Cat# AF-294-NA), and the phosphor-Met antibody was purchased from Cell Signaling (Cat# 3077). Mucicarmine staining kit was purchased from Dako Cytomation (Cat# SL016).

### Bioluminescent Imaging

Mice were injected with 250 mg of Luciferin (Invitrogen) in PBS and anesthetized with Isoflurane. The mice were then transferred into the chamber of the IVIS Spectrum imaging system (Caliper) under anesthesia. Mice were typically imaged 8 to 10 minutes after injection. The colon model chimeras were typically imaged for 5–10 min under the maximum sensitivity setting.

### Tumor cell processing and propagation

Dissected colon tumors were rinsed in PBS, then either cut into 3 mm cubes for implantation or digested with collagenase to single cell for injection.

For subcutaneous propagation, 100 µL of digested tumor cells in Matrigel were injected into the subcutaneous space on the flank. Alternatively, 3 mm tumor chunks were implanted with a trocar under anesthesia.

For kidney capsule propagation, recipient mice (either NOD or ICR Scid) were anesthetized and shaved. A small incision was made on the back to the right of the spine directly below the rib cage line. The kidney was scooped out of the opening and held carefully with a pair of large blunt ended forceps. 10–20 µL of tumor cells in Matrigel were drawn into a Hamilton syringe with a 27 gauge needle and the needle was carefully inserted just under the kidney capsule. Cells were slowly injected to form a visible bleb. The needle was carefully removed 10 seconds after injection. Alternatively, a 3 mm tumor chunk was inserted into a small hole created with watchmaker’s forceps. The kidney was returned followed by suturing of the abdominal wall and skin.

For cecal sub-mucosal propagation, recipient mice (either NOD or ICR Scid) were anesthetized and shaved. A small incision was made on the abdominal body wall slightly to the left of the midline between the genitals and the rib cage. The cecum was gently pulled out using blunt forceps and laid flat on the abdomen. 10–20 µL of tumor cells in Matrigel were drawn into a Hamilton syringe with a 27 gauge needle. With the intestine held taut by forceps, the needle was carefully inserted just under the cecum capsule between the two visible blood vessels. Cells were slowly injected to form a visible bleb. The needle was carefully removed 10 seconds after injection. The cecum was returned followed by suturing of the abdominal wall and skin.

For seeding of tumor cells into the liver, recipient mice were anesthetized. A small incision was made on the ventral side of the mouse through the skin and muscle layer. The spleen was gently pulled out using blunt forceps and laid on sterile saline moistened gauze. Tumor cells in 0.2 mL of media were injected with a 25–27 g needle into the lower pole of the spleen in 2 minutes. The infusion site (spleen pole) was ligated. The needle was carefully removed 10 seconds after injection. The spleen was then resected followed by suturing of the abdominal wall and skin.

### Detection of spontaneous metastasis

50–100 thousand collagenase digested CB42 tumors cells were injected into the subcutaneous space. When the tumors reach the size of 400–800 mm^3^, the tumor lump as well as the skin over the lump was resected under anesthesia. The skin was then stapled back together and the mice were treated with analgesics for the next 48 hours. These mice were carefully monitored for signs of morbidity and/or weight loss, which indicate metastasis of tumor cells to vital organs.

### Array-CGH profiling and analyses

Genomic DNA processing, labeling and hybridization to Agilent CGH 60-mer oligo arrays were performed as per the manufacturer’s protocol (http://www.home.agilent.com/agilent/home.jspx). Briefly, DNA was isolated from snap frozen tumor tissue or fresh ES cells using the Gentra Puregene Kit following kit instructions but with double the recommended volume of reagents. aCGH was performed by Asuragen using the Agilent 244 K mouse genome aCGH chip. Each tumor DNA sample was hybridized against the corresponding ES cell DNA as the reference. Labeled DNAs were hybridized onto 244 K microarrays (detailed feature information for each at http://www.agilent.com). Fluorescence ratios of scanned images were normalized, and copy number profile was generated by Circular Binary Segmentation, which determines significance of change points in raw data through permutation [43].

Definition of minimal common region (MCR) was described previously [44], [45]. Briefly, a ‘segmented’ data set was determined from uniform copy number segment boundaries, then replacing raw log2 ratio for each probe by the mean log2 ratio of the segment containing the probe. Thresholds for copy number alterations were set at log2 = +/0.6. MCRs required the width less than 10 Mb.

### In vivo efficacy studies

The MET inhibitor crizotinib, the CDK4/6 inhibitor PD0332991, and the MEK inhibitor PD0325901 were synthesized by Sundia Meditech (Shanghai, China). The activity of crizotinib was verified by inhibition of MET phosphorylation in U87MG cells using MSD assay (MS6000 Phospho-, Total MET Whole Cell Lysate Kit, Mesoscale, Cat# K15126D-2). The activity of PD0332991 was verified by inhibition of Rb phosphorylation in MCF7 cells using Western blot. The activity of PD0325901 was verified by inhibition of Erk1/2 phosphorylation in H441 cells as well as CB42 cells using Western blot. For in vivo studies, crizotinib was formulated freshly daily in 0.5% methylcellulose and mice were dosed daily with 50 mg/kg of crizotinib by oral gavage. PD0332991 was formulated freshly daily in 0.5% methylcellulose and mice were dosed daily with 125 mg/kg of PD0332991 by oral gavage. PD0325901 was formulated freshly daily in 0.5% hydroxypropylmethyl-cellulose/0.2% Tween 80 and mice were dosed PO daily with 5 mg/kg or 15 mg/kg of PD0325901.

## Supporting Information

Table S1 **Detailed information of tumors tested for propagation.**
(XLSX)
